# Physical Education

**Published:** 1900-06-02

**Authors:** 


### The Hospital

A Journal of

The Medical Sciences and Hospital Administration.

Vol. XXVIII.—No. 714.] Edited by Sir Henry Burdett, K.C.B., and Solomon C. Smith, M.D., M.R.C.P. [June 2, 1900.

### Physical Education.

Among the national questions which the events of the war in South Africa seem likely to bring into prominence, few can be more important than that of improved physical education. It is an old military dictum, which has been attributed to every general of repute from Hannibal to Wellington, and which will no doubt soon be repeated to us on the authority of Lord Roberts or Lord Kitchener, that victories are won more by the legs of soldiers than by their arms, in whichever of its two principal senses the last word may be employed. There is still higher sanction for the assertion that, when a strong man armed keepeth his palace, his goods will be at peace until a stronger than he cometh; and it will be a matter of no small speculative and of still more practical concern to observe whether the people of this country have as yet profited sufficiently by the results of the Education Acts to be able to recognise their true interests, and to be prepared to make sacrifices for their realisation. If a country is to be well governed it must govern itself, using politicians as implements, but not blindly trusting to them as guides; and the time has come at which strong and definite pressure should be applied by public opinion, for the purpose of rendering our schools efficient agents for securing that the bodily development of future generations shall be duly cared for by all who are placed in positions to aid in bringing about such a result. Just as the Navy League has already been a powerful influence for good in the service upon which its efforts are concentrated, and as a similar organisation is projected to secure that even the most cherished *alumni* of the Staff College shall be made to understand the difference between war and skittles, so an equal application of energy is needed in order to bring home to all whom it may concern that the rising generation of Englishmen must be rendered shrewd if possible; but, if not shrewd, at all events strong, and capable, under a proper leader, of fighting the battles of their country wherever, and by whomsoever, her rights or her interests may be assailed.

As an element in the maintenance of national health, there can be no doubt that the physical training of the young should occupy a very conspicuous place. Our enlarged knowledge of the causes of disease, and our power of referring its different forms to this or that micro-organism, have left untouched the much older knowledge that these causes are to a great extent assisted in their operation by independent conditions. It is in the narrow and imperfectly expanded chest that the bacillus of tubercle finds its most appropriate habitation, and in which it most freely exercises its powers of multiplication and destruction. It is the under-nourished brain, fed with imperfectly aërated blood by a feeble heart and by defective lungs, which furnishes the most appropriate centre for volitions applied to the gratification of criminal impulses. It is impossible not to observe, moreover, that the defective bodily development which physical education might correct seldom becomes apparent until the age for schooling has been reached. The little ragamuffins who play and tumble about in the poorer streets of our great cities are, with few exceptions, strong, healthy, and active enough; and it is usually at a later period that their development becomes blighted or arrested. It is the hobbadehoy who falls into habits destructive of the health and symmetry of the man; habits which convert a promising child into a slouching loafer; and it should be the business of our schools to preserve us from the sloucher no less than from the dunce. A boy who has been taught to hold up his head, to straighten his knees, to fill his chest, and to walk as if he were going somewhere with an object in view, will certainly gain in self-respect and efficiency as a human creature; and it is not likely that he would suffer even if measured by the narrow standard of an examiner. The contests of Olympia had no small influence in promoting the intellectual development of Greece; and, many years ago, the late Mr. Paget, M.P., tried an interesting experiment in the village schools on his estate at Ruddington, in Nottinghamshire. He attached a sufficient plot of garden ground to the schools, and divided the scholars as nearly as possible into two equal groups. One of these groups remained in the schoolroom, and was kept to school tasks for the full ordinary hours; while the other group spent only half-time in the school, and devoted the other half to steady work in the garden under proper instruction and supervision. At the end of six months, the "whole-timers" were utterly distanced by the gardening children, who were not only far and away first in school work, but first also in bodily strength and capability. Our national horizon is far from being cloudless, and it is our duty to provide for the health and strength of the coming generation. Let us shorten the time during which our children are imprisoned in a vitiated atmosphere, and devote the period that is gained to systematic endeavours to render their bodily frames fitted for that burden of empire which it is our destiny to bear.

